# Whole Fabric‐Assisted Thermoelectric Devices for Wearable Electronics

**DOI:** 10.1002/advs.202103574

**Published:** 2021-11-05

**Authors:** Yue Hou, Yang Yang, Ziyu Wang, Zhaoyu Li, Xingzhong Zhang, Brandon Bethers, Rui Xiong, Haizhong Guo, Hongyu Yu

**Affiliations:** ^1^ Department of Mechanical and Aerospace Engineering The Hong Kong University of Science and Technology Kowloon Hong Kong SAR 999077 P. R. China; ^2^ Department of Mechanical Engineering San Diego State University 5500 Campanile Drive San Diego CA 92182 USA; ^3^ The Institute of Technological Sciences Wuhan University Wuhan 430072 P. R. China; ^4^ Key Laboratory of Artificial Micro‐ and Nano‐Structures of Ministry of Education School of Physics and Technology Wuhan University Wuhan 430072 P. R. China; ^5^ Key Laboratory of Material Physics Ministry of Education School of Physics and Microelectronics Zhengzhou University Zhengzhou 450052 P. R. China; ^6^ Collaborative Innovation Center of Light Manipulations and Applications Shandong Normal University Jinan 250358 P. R. China

**Keywords:** conductive electrodes, elastic fabrics, electrical skin, flexibility, thermoelectric devices, wearable electronics

## Abstract

Flexible thermoelectric generators (f‐TEGs) have demonstrated great potential in wearable self‐powered health monitoring devices. However, the existing wearable f‐TEGs are neither flexible enough to bend and stretch while maintaining the device's integrity with a good TE performance nor directly compatible with clothes materials. Here, ultraflexible fabric‐based thermoelectric generators (uf‐TEGs) are proposed with conductive cloth electrodes and elastic fabric substrate. The patterned elastic fabric substrate fits the rigid cuboids well, together with serpentine structured cloth electrodes, rendering uf‐TEG with excellent integrity and flexibility, thereby achieving a highly functional TE performance when strain reaches 30% or on arbitrarily shaped heat sources. The uf‐TEGs show a large peak power of 64.10 *μ*W for a temperature difference of 33.24 K with a high voltage output of 111.49 mV, which is superior compared to previously reported fabric‐based TEG devices, and it is still functional after the water immersion test. Besides the energy harvesting function, with both the temperature sensing ability and the touch perception, this uf‐TEG is demonstrated as the electrical skin when mounted on a robot. Moreover, due to the wind‐sensitive performance and self‐power ability, the uf‐TEGs are assembled on cloth as wearable health and motion monitoring devices.

## Graphical abstract

An ultraflexible whole‐fabric assisted thermoelectric generator (uf‐TEG) with thermoelectric cuboids, elastic fabric as assembling material, and conductive flexible cloth‐based electrodes is presented here. The uf‐TEG can be applied in different applications such as electrical skin (temperature sensing and touch perception) and human health and motion monitoring (respiration rate, finger gesture, and steps counting).

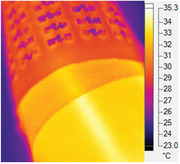

## 1 Introduction

In the past decade, technological advancement has sped up the development of wearable electronic devices for health and motion monitoring. To substitute the large battery for these wearable electronic devices and acquire a sustainable energy source, the idea of generating power from the human body itself has bred various devices, such as thermoelectric generators (TEGs) and triboelectric nanogenerators (TENGs).^[^ ^1^, ^2^, ^3^ ^]^ Among them, TEGs can generate power where the thermal gradient exists in TE materials (Seebeck effect), which ensures a stable power output when put on the body. As heat sources on the human body have nonplanar surfaces, softening the TEGs and achieving flexibility to have close contact with the heat source is critical for harvesting heat. Nowadays, pioneer works on flexible TEGs focus on three types of materials, from 1D to 3D, namely TE fibers or nanowires,^[^ ^4^, ^5^, ^6^, ^7^, ^8^ ^]^ TE thin‐film based on ink processing methods,^[^ ^9^, ^10^, ^11^, ^12^, ^13^, ^14^ ^]^ and conventional TE bulk materials.^[^ ^15^, ^16^, ^17^, ^18^, ^19^, ^20^, ^21^ ^]^ To achieve good flexibility, scientists have developed TE fibers woven or sewed on textiles. For instance, Lund et al. fabricated flexible TEGs with an output power of 1.2 µW (Δ*T* = 65 K) by sewing poly(3,4‐ethylenedioxythiophene) polystyrene sulfonate (PEDOT: PSS) coated threads and silver‐plated polyamide threads on thick wool fabric.^[^ ^4^ ^]^ A similar sewing process can also be found in Ding et al., where they used gelation extrusion strategy to fabricate p/n TE fibers and woven them onto the textile. The output power for the whole weaved TE textile is at pW level (Δ*T* = 5–20 K).^[^ ^6^ ^]^ Although textile renders good flexibility, the unsatisfied TE converting performance from the fiber‐based materials and the hand sewing process limited the TEGs in large‐scale applications. For 2D thin‐film‐based TEG devices, thin films were usually scratched on substrates such as paper or polyimide (PI) to obtain a certain level of bendability. But the in‐plane assembled TE pairs restrict the direction of the along film surface.^[^ ^14^ ^]^ To solve this problem, researchers used specific kirigami structures to fold the plane substrate and help the former in‐plane device perpendicular to the surface of the heat source.^[^ ^15^, ^22^, ^23^ ^]^ However, the performance is still not ideal, and the device's stretchability was severely affected after folding. Furthermore, the thermoelectric effect of both fiber and 2D films is weak compared with bulk materials. Therefore, researchers are always looking at solutions on flexible devices using 3D bulk TE materials. TE cuboids, which are made of Bi‐Te and Sb‐Te based materials are an option due to their high performance at room temperature. The high figure of merit (ZT value) of these inorganic compounds guarantee the excellent energy converting performance of the thermoelectrical device. To endow the TEGs of rigid cuboids with a certain stretchability level, Fukuie et al. chose an origami‐like PI substrate and assembled the TE cubes on it, thereby achieving 20% stretching deformation.^[^ ^17^ ^]^ Lee et al. proposed another method to soften the TEGs by using silver nanowires‐based stretchable interconnects to connect paralleled cuboids to substitute traditional rigid copper electrodes, while the intermediate regions between cuboids were infiltrated with polydimethylsiloxane (PDMS) to hold the cuboids.^[^ ^18^ ^]^ However, there exist detaching issues between soft PDMS and the rigid TE cuboids in a large bending or stretching situation. Besides, the PDMS material itself is incompatible with fabric when considering wearable TEGs.

Herein, we present a ultraflexible fabric‐assisted TEGs (uf‐TEGs) combining Bi‐Te and Sb‐Te based TE cuboids, elastic fabric as packaging material, and conductive flexible cloth‐based electrodes to address the above challenges. Cuboids with high TE conversion performance were assembled with elastic fabric for holding cuboids in position to acquire excellent device function. Conductive polyester fiber‐based electrodes were laser‐cut as the serpentine structure to reduce stiffness of electrodes and better fit the fabric substrate under large deformation. Using a scalable pick‐and‐place semi‐automated process, uf‐TEGs were fabricated with different sizes and applicable to heat sources in different areas. The flexibility and stretchability of uf‐TEGs that are achieved from the all‐fabric design conform to the morphological characteristics of human skin, enabling it as a wearable energy harvester. Furthermore, together with functions of temperature sensing and touch perceptions, the uf‐TEGs demonstrate a perfect candidate for electrical skin (e‐skin). Other applications for wearable health and motion monitoring are also included here to show its practicability.

In previous research, the flexible printed circuit board with PI substrate is one option for connecting TE cuboids.^[^ ^15^ ^]^ However, the PI substrate is not soft and flexible enough (show little bendability in one direction) to cover heat sources with complex surface structures, and it cannot be assembled with clothes and stretched. Others used silicone materials (PDMS or Exoflex) to assemble or package the TEGs,^[^ ^16^, ^18^ ^]^ but the soft silicone materials will easily detach from the rigid cuboids after several stretching or bending circles and affect the device's integrity. Besides, the PDMS packaged devices are impossible to combine with clothes. From the perspective of conformal contact and better compatibility with clothes, the uf‐TEG demonstrates its advancement in 1) being able to be directly fabricated on clothes with washability, 2) advanced flexibility and deformability, and 3) showing no detach issue happened in the connected region of elastic fabric and cuboids when compared with the previous research using rigid TE cuboids.

## 2 Design of the Semi‐Automated Process for Fabric‐Combined TEG

**Figure 1:**
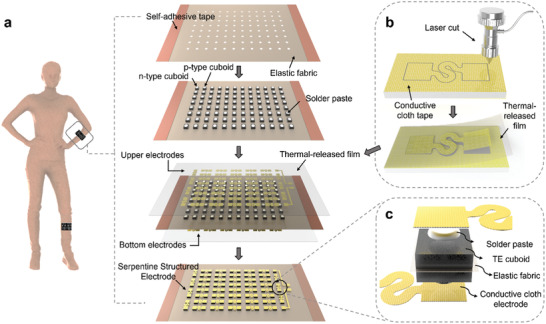
Design of the uf‐TEGs. Left to right: a) Schematic diagram of the fabrication process for the uf‐TEG of the 12 × 8 array (48 pairs of p–n couples). b) Pick‐and‐place method using laser‐cut machining and the thermal‐released film. c) Configuration for one connected TE cuboid.

The uf‐TEGs consist of rigid TE cuboids (p‐type: Bi_0.5_Sb_1.5_Te_3_/n‐type: Bi_2_Te_2.7_Se_0.3_), elastic fabric substrate, and conductive cloth tape electrodes. **Figure** **1**a shows the configuration of the uf‐TEGs (48 pairs with the designed array size of 104.5 mm × 71.0 mm), which is highly compatible with our daily wears. The uf‐TEGs were fabricated and assembled through a semi‐automated process, including laser cutting and the pick‐and‐place assembling process (detailed information in Figure S1, Supporting Information). Targeted primarily at the challenge of the alignment during transferring electrode arrays, we promoted a novel pick‐and‐place method illustrated in the diagram of Figure 1b. First, the TE cuboids were placed on a temporary hard substrate with predesigned gaps for p‐type and n‐type cuboids arrangement. The elastic fabric with laser‐cut holes was then placed on top of the hard substrate and pushed down to allow cuboids to sit in the holes of the fabric. Then, a layer of solder paste was scratched on each of the cuboids through a patterned polyethylene terephthalate (PET) mask. Second, the serpentine structured electrodes were patterned on the conductive cloth tape through a laser‐cut process, and the electrode pattern was further proved to be the most suitable one considering the impact of fill factor (the ratio of the total area of the TE elements to the area of the whole device) (Figure S10, Supporting Information). Then, a piece of PET thermal‐released film was applied to pick up all serpentine structures from the conductive cloth and then transfer them to the top of the TE cuboids array (Figure 1b). During this process, the alignment with the TE cuboids was done by aligning thermal‐released film with the elastic fabric, and the relative location between every electrode was consistent with the original cut pattern. The whole sample was then put on a hot plate at 180 °C for 5 min for solder bonding. Then, the cuboids, elastic fabric, conductive cloth electrodes, and thermal‐released film were detached from the temporary hard substrate and the bottom electrodes were assembled through the same method. Finally, a heat gun of 120 °C was used to heat the film surfaces (top and bottom electrodes), and the PET thermal‐released films were automatically shrunk and separated from the electrode arrays. As shown in Figure 1c, for every single TE cuboid (5.0 mm ×  5.0 mm ×  3.0 mm) (length ×  width ×  sample thickness), both upper and bottom sides were connected firmly to the polyester fiber‐based electrodes (thickness ≈ 0.07 mm) through a thin layer of solder paste.

## 3 Material Properties and Output Performance of uf‐TEG

**Figure 2:**
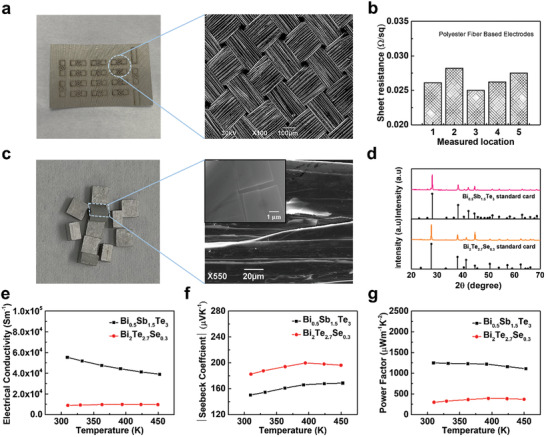
Material properties of conductive polyester fiber‐based electrodes and TE cuboids. a) The photoimage and the SEM image of the polyester fiber‐based electrodes. b) The sheet resistance of the polyester fiber‐based electrodes measured in five different locations. c) The photoimage and the SEM image (cross‐section view) of the TE cuboids. d) XRD patterns of Bi_0.5_Sb_1.5_Te_3_ and Bi_2_Te_2.7_Se_0.3_. Temperature‐dependent e) electrical conductivity, f) absolute Seebeck coefficient, and g) power factor of both Bi_0.5_Sb_1.5_Te_3_ and Bi_2_Te_2.7_Se_0.3_.

The scanning electron microscope (SEM) image of **Figure** **2**a clearly demonstrates the surface structure of the electrode with woven polyester fibers. The sheet resistance *R*_S_ of conductive cloth was tested at five different locations using a four‐point‐probe mapping system with an average value of 0.0266 Ω sq^−1^(Figure 2b). Subsequently, the bulk resistivity of the uniform cloth film can be derived from *ρ*  = *R*_S_  × *t*, where *t* is the thickness and the resistivity of the conductive cloth is ≈1.86 × 10^−6^Ω m. For the TE elements Bi_0.5_Sb_1.5_Te_3_ and Bi_2_Te_2.7_Se_0.3,_ the sintered blocks' nanolayered properties after hot‐pressing were demonstrated in the SEM images in Figure 2c. The X‐ray diffraction (XRD) patterns detected from Bi_0.5_Sb_1.5_Te_3_ and Bi_2_Te_2.7_Se_0.3_ were well matched with their standard card data, identifying the crystallographic structure of the perspective materials (Figure 2d). Figure 2e,f shows the electrical conductivity (*σ*) and Seebeck coefficient (*s*) of the Bi_0.5_Sb_1.5_Te_3_ and Bi_2_Te_2.7_Se_0.3_ cuboids versus temperature from 304 to 453 K, respectively. Based on *σ* and *s*, the power factor (PF) of two TE materials can be calculated as $\mathrm{PF}\;=\frac{s^{2}}{\rho }\;=s^{2}\;\cdot \sigma$, which is also provided in Figure 2g as 1249.81 *μ*W m^−1^ K^−2^ at 309.36 K for Bi_0.5_Sb_1.5_Te_3_ and 298.01 *μ*W m^−1^ K^−2^ at 310.05 K for Bi_2_Te_2.7_Se_0.3_. High PF of materials ensures high ZT values ($\mathrm{ZT}\;=\frac{\mathrm{PF}\times T}{\kappa }\;$, where *k* stands for the thermal conductivity), guaranteeing a good performance for the uf‐TEGs.^[^ ^24^ ^]^

**Figure 3:**
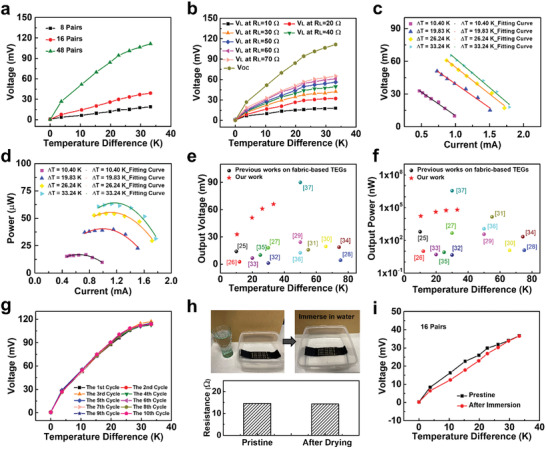
The performance of the uf‐TEGs. a) The output voltage of uf‐TEGs (8, 16, and 48 pairs) at different Δ*T*. b) The output voltage of uf‐TEG (48 pairs) at different Δ*T* with and without load resistance (load resistance from 10 to 70 Ω). c) The output voltage of uf‐TEG (48 pairs) as a function of electrical current. d) The output power of uf‐TEG (48 pairs) as a function of electrical current. e) Comparison of the output voltage of the uf‐TEG (48 pairs) with other fabric‐based TEGs. f) Comparison of the output power of the uf‐TEG (48 pairs) with other fabric‐based TEGs. g) Cyclic heating tests of uf‐TEGs (48 pairs). h) Immersion test of uf‐TEG (eight pairs) and the device resistance before and after the immersion test. i) The output voltage of uf‐TEG (16 pairs) as a function of increasing temperature before and after immersion test.

As shown in **Figure** **3**a, the output performances of the uf‐TEGs with different TE pairs (8, 16, and 48 pairs) were measured. The output voltage under an increasing temperature difference (Δ*T*) was collected via a data acquisition card (National Instruments, NI) (testing setup is provided in Figure S2, Supporting Information). Both the open‐circuit voltage (*V*_OC_) of uf‐TEG (48 pairs) and the output voltage (*V*_L_) with the load resistance (*R*_L_) varying from 10 to 70 Ω were collected in Figure 3b. The *V*_OC_ reached a value of 111.49 mV under a temperature difference (Δ*T*) of 33.24 K, which was superior within the reported flexible TEG devices (Figure 3e),^[^ ^25^, ^26^, ^27^, ^28^, ^29^, ^30^, ^31^, ^32^, ^33^, ^34^, ^35^, ^36^, ^37^ ^]^ and a large $\frac{\Delta V}{\Delta T}$ also ensured a more distinguishable signal when applied to e‐skin applications mentioned in the latter section. The relations of voltage–current and power–current were obtained when the temperature difference was 10.40, 19.83, 26.24, and 33.24 K, and their fitting curves were also plotted in Figure 3c,d, respectively. When the temperature difference reached 33.24 K, the uf‐TEG achieved the maximum power of 64.10 *μ*W, which was excellent within previously reported fabric‐based TEGs (Figure 3f). ^[^ ^25^, ^26^, ^27^, ^28^, ^29^, ^30^, ^31^, ^32^, ^33^, ^34^, ^35^, ^36^, ^37^ ^]^ To test the long‐term stability, the uf‐TEG (48 pairs) was placed on the hot plate for the heating process and then using an electric fan to speed up the cooling process, and Figure 3g illustrated the performance for ten repeated heat cycles, which has demonstrated a good stability with the largest *V*_OC_ variation of 4.09% when Δ*T* = 33.24 K. Furthermore, the water resistance of the uf‐TEG (16 pairs) was also tested by immersing the uf‐TEG into the water for 30 min followed by a drying process inside an oven for 12 h at 70 °C (Figure 3h), the resistance of the uf‐TEG dropped slightly from 14.6 to 14.3 Ω when the sample was dried. The *V*_OC_–Δ*T* curves of uf‐TEG were also measured before and after the immersion test. The results in Figure 3i demonstrate a comparable TE performance of uf‐TEG that dried after the immersion test, demonstrating the reliability of our device and the potential use in cloth‐assembled e‐skin applications.

## 4 Flexibility Testing of uf‐TEG

**Figure 4:**
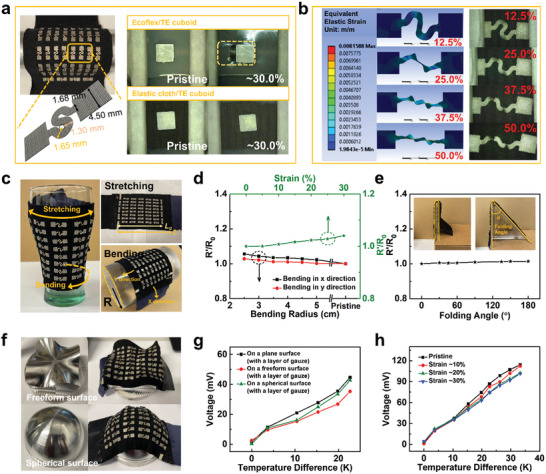
Electrode design and flexibility testing for the uf‐TEG. a) Design parameters of the serpentine structured electrode and the comparison test under a strain of 30% for the interface of TE cuboid/Ecoflex and TE cuboid/elastic fabric. b) FEA simulation and the experimental results of deformation for single serpentine structured electrode when the strain reached 12.5%, 25%, 37.5%, and 50%, respectively. c) Representative images for stretching, bending, and folding situations. d) The resistance variation (*R*’/*R*_0_) during the folding tests (the folding angle changed from 0^○^ to 360^○^ with 90^○^ interval in between) for uf‐TEGs with 48 TE element pairs. e) The resistance variation (*R*’/*R*_0_) during the stretching tests (under the tensile strain from 0% to 50%), bending tests (with bending radius variated from 1.0 to 3.0 cm, 0.5 cm interval in between) for uf‐TEGs with 48 TE element pairs. f) Demonstrations of heat collection on aluminum blocks with a freeform surface and a spherical surface. g) The output performances for uf‐TEG with 48 TE element pairs on arbitrary surfaces. h) The output performances for uf‐TEG with 48 element pairs under a strain of 10%, 20%, and 30%, respectively.

The uf‐TEGs demonstrated high deformability owing to the elastic fabric substrate and highly stretchable conductive‐cloth‐based electrodes. As demonstrated on the right of **Figure** **4**a, comparison tests were conducted to verify the stability of the interface between the rigid cuboid and its linked material (Ecoflex and elastic fabric). Although prior researches from other groups chose soft silicone materials to connect the cuboids,^[^ ^15^, ^38^ ^]^ the mismatch and debonding between the rigid and soft material leads to the gap on the border (pointed out in a yellow rectangle). Compared with silicone material, our elastic cloth with laser‐cut holes demonstrated good compatibility with a firm holding of the TE elements and no evident gap under a strain of 30.0%, showing its superior stable cuboid holding capability over the silicone materials connected device.

For every single electrode, the serpentine structure with a trace width of 1.30 mm has rendered the electrode with over 50.0% of stretchability without fracture (Figure 4a). This type of structure has been widely used for many other flexible devices due to its lower stiffness under the stretching process.^[^ ^39^, ^40^, ^41^ ^]^ Finite element analysis (FEA) simulation was used to simulate the electrode deformation under the strain of 12.5%, 25.0%, 37.5%, and 50.0%, respectively. As shown in Figure S7c in the Supporting Information, the largest stress is located on the electrode's inner parts. As the elastic strain increased, the maximum stress was concentrated on the top and bottom part of the middle serpentine structure, explaining the electrode deformation of these two parts during the stretching process (Figure 4b). The small resistance variations during the stretching, bending, and twisting for every single electrode were verified as shown in Figure S7a in the Supporting Information, which paved the way for the stable TE performance after assembling with TE elements on cloth substrate. As shown in Figure 4c, to better suit arbitrary surfaces such as arms or joints of the human body or angular regions of robots, the fabricated uf‐TEG (48 pairs) was also tested under different stretching and bending situations. When the uf‐TEG was stretched to 30.0%, the corresponding resistance variation was 4.06% (green line in Figure 4d). When the uf‐TEG was bent on acrylic molds with different bending radii from 2.5 to 5.0 cm, with 0.5 cm intervals in between in both *x* and *y* directions, the resistance variations were relatively small as they increased by 5.55% and 2.82%, respectively, at their largest bending degree (bending radius of 2.5 cm) (black and red lines in Figure 4d). In addition, the uf‐TEG was folded at its middle with a relatively small resistance variation of 1.42% at the folding angle of 180° (Figure 4e). To further demonstrate its flexibility, two aluminum blocks with a freeform surface and a spherical surface, respectively, were machined and used as the heat source for energy harvesting tests (Figure 4f). The relation of *V*_oc_–Δ*T* was measured when uf‐TEG was placed on the freeform surface, the spherical surface, and the plane surface successively while the aluminum block was heating up on a hot plate. During this test, the uf‐TEG was distorted to fit the substrate at its greatest extent, and a layer of gauze was placed in between to avoid a short circuit. As shown in Figure 4g, the uf‐TEG was still functional with degradation of 4.44% and 20.01% with the spherical and freeform surfaces, respectively, when compared with the plane surface. Notably, the relation of *V*_oc_– Δ*T* was also measured for uf‐TEG under the strain of 10.0%, 20.0%, and 30.0%, respectively (Figure 4h). The results further proved the stability of uf‐TEG with a relatively high Voc of 101.44 mV at Δ*T* of 33.24 K under a strain of 30.0%.

## 5 Applications of uf‐TEG as E‐Skin and Other Wearable Monitoring Devices

Fabric‐based TEGs have been widely studied and demonstrated for skin heat harvesting.^[^ ^42^, ^43^, ^44^, ^45^ ^]^ However, few of these fabric‐based TEGs offered practical applications after turning the heat energy into electricity. In previous sections, the good flexibility and unprecedented large output performance of the uf‐TEG when compared with other fabric‐based TEGs have been demonstrated. This suggests that heat energy can be reused for sensing applications with good performance.

**Figure 5:**
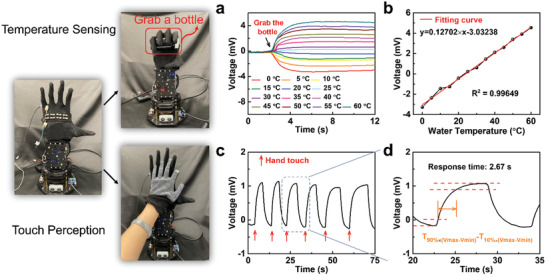
Uf‐TEG based potential e‐skin application with the functions of temperature sensing and touch perception. a) Temperature sensing performance for uf‐TEGs of eight pairs. b) Experimental result and fitting curve for *V*_output_–*T*_water_. c) Touch perception test with uf‐TEGs. d) Response time for touch perception test.

Human skin, densely covered with the sensory nerve fibers, serves as the sensing function for outside heat, touch, etc. Here, thermoelectric ability endows the uf‐TEG with the functions of temperature sensing and touch perception ability. Together with the device's good flexibility and stretchability that mimic skin morphology, there is great potential for uf‐TEG to be applied as e‐skin in the field of robotic engineering. As shown in **Figure** **5**, the uf‐TEG (eight pairs) was assembled on a glove and worn on a robotic hand. The voltage output was monitored throughout the whole process of grabbing the bottle with different water temperature (*T*_water_) that variated from 0 to 60 °C with 5 °C intervals in between. The output voltage (*V*_output_) suddenly increased (25 °C ≤  *T*_water_ ≤  60 °C) or decreased (0 °C ≤  *T*_water_  ≤  20 °C) when the uf‐TEG touched the bottle (Figure 5a). After the *V*_output_ became stable, the relationship between *V*_output_ and *T*_water_ was collected in Figure 5b with good linearity (coefficient of determination (*R* ^2^) ≈ 0.99649). Apart from sensing the temperature, the uf‐TEG could also be used for touch perception. As is shown in Figure 5c,d, the robot hand with uf‐TEG can detect the touch of the human hand with a fast response of ≈ 2.67 s, which further imitated the biological function of the human skin.

**Figure 6:**
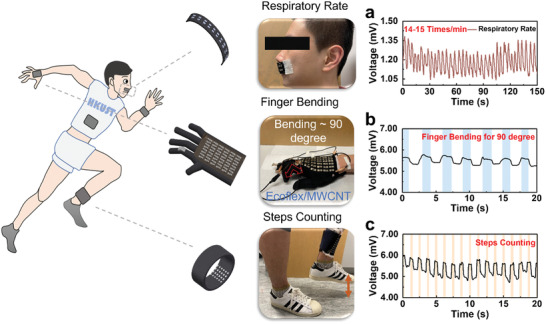
Uf‐TEG based wearable devices for human motion monitoring. a) Human respiratory rate (RR) detection using a small uf‐TEG patch (eight pairs). b) Finger bending detection with uf‐TEG based glove (48 pairs) on hand. c) Steps counting based on the uf‐TEG wristband during a stepping process.

Instead of being used as e‐skin, uf‐TEGs can also be incorporated into everyday clothing such as cloth, gloves, and wristbands and used for health or motion monitoring. As the output voltage increased with the air velocity on the cold side of uf‐TEG (Figure S4, Supporting Information), a small uf‐TEG patch (4 × 2 pairs) was placed in the region between the nose and mouth to detect the nose expiratory rate. In **Figure** **6**a, the expiratory rate from the nose for the volunteer in the static state was around 14–15 times min^−1^. Other applications for human motion monitoring are demonstrated in Figure 6b,c. Here, the uf‐TEGs served as a voltage source to supply power for electrical resistance variation indication. The flexibility of the uf‐TEG ensured an excellent contact with the skin surfaces and gave a voltage output of ≈ 5.7 mV for uf‐TEGs with 48 TE pairs. By supplying a constant voltage for a strain‐sensitive conductive elastomer (Ecoflex/Multi Walled Carbon Nanotubes) sewed onto the glove's knuckle part (Figure 6b) or for a piezoresistive sensor assembled under a sock (Figure 6c), the voltage output variation represented the finger bending and the stepping process, respectively, due to the variated load resistance. Other experiments for finger tapping and more experimental details are provided in the Supporting Information (Figures S4–S7, Supporting Information).

## 6 Conclusion

Using a semi‐automated fabrication process that includes laser‐cut and pick‐and‐place methods, cloth‐based conductive electrodes and elastic fabric have been assembled on a large scale with rigid TE cuboids in a good alignment. TE cuboids with a high power factor were chosen for this uf‐TEG to ensure a well‐performing device. Compared with other fabric‐based TEGs, the uf‐TEGs achieved an unprecedentedly high output voltage and power of 111.49 mV and 64.10 *μ*W, respectively, when the Δ*T* turned to 33.24 K. Moreover, the devices show good flexibility as they can be stretched, bent, or folded to suit surfaces such as a hemisphere and an uneven freeform surface with suitable attachment and almost unaltered performance. High TE performance and the devices' good flexibility make the uf‐TEG a perfect candidate for building a self‐power sensing system.

Traditional TEGs also employ rigid TE cuboids, compared with them, our uf‐TEG demonstrates its advancements in

1) Ultra flexibility and deformability to suit heat sources with complex geometry.
2) Compatibility with our everyday clothes.
3) Enhanced cuboids connection with no detaching issue by using the elastic fabric substrate.

In this paper, uf‐TEGs are demonstrated with practical sensing applications, including electrical skin and wearable health and motion monitoring devices. When used as an e‐skin, both the biological function (temperature sensing and touch perception) and its morphology conform to human skin. Besides, the uf‐TEG can also be applied as wearable health and motion monitoring device, since it can detect, amongst other things, the nose respiration rate, finger bending, and steps counting. However, the energy harvested using uf‐TEGs is not enough to fulfill a fully self‐powered sensing system at the current stage. This is hindered by the devices' internal resistance, which causes energy waste on internal power consumption. Therefore, future efforts will be made to improve the conductivity of the cloth electrode.

To conclude, the fabricated uf‐TEGs provide an excellent way to harvest heat from randomly shaped heat sources and reuse the waste heat energy in applications with practical functions, providing inspiration for the future development of fully thermoelectrically self‐powered fabric‐based devices.

## 7 Experimental Section

### Sample Preparation

The serpentine structured electrode arrays were first designed with a line width of 1.30 mm and the pad side length of 5.00 mm in AutoCAD. The polyester conductive tape (total thickness with the paper substrate: ≈130 µm, without the substrate: ≈70 µm, conductive component: electroplated Cu‐Ni alloy) was purchased from Shenzhen Shunzhi Technology. A laser‐cut machine cut the polyester conductive tape under the power of 7.6 W (± 0.1 W) and a speed of 10 mm s^−1^. The energy and cut speed ensure that the process cuts off the upper polyester conductive tape without totally cutting off the based paper substrate.

TE cuboids of Bi_0.5_Sb_1.5_Te_3_ (p‐type) and Bi_2_Te_2.7_Se_0.3_ (n‐type) were purchased from Hubei Sagreon New Energy Technology Company, Ltd and the size of the cuboids was custom‐made with the size of 5 mm  ×  5 mm  ×  3 mm. To conduct the pick‐and‐place process, transparent PET thermal‐released film was purchased from Crown Electronics with a releasing temperature of 120 °C. (Details of the fabrication appear in Text S1 and Figure S1, Supporting Information.) Low‐temperature lead‐free solder paste (ECO SOLDER Sn_42_Bi_58_) with a melting point of 138 °C was purchased from Senju Metal Industry Co., Ltd. During the soldering process, the temperature was set as 180 °C and the soldering time was around 5 min.

Ordinary elastic thread fabric of pure cotton was purchased online and the elastic fabric was not strictly limited to this specific type. The elastic fabric was laser cut to form a pattern of circular hole arrays (more details of the fabrication appear in Text S1 and Figure S1, Supporting Information).

### Materials Characterization

SEM was used to characterize the surface morphology of the polyester conductive tape and TE cuboids. In the flexibility test, bending molds with different bending radius (1–3 cm, 0.5 cm interval in between) were prepared from the laser‐cut acrylic workpieces. Acrylics tubes with different bending radius (2.5–5 cm, 0.5 cm interval in between) were prepared for bending tests of uf‐TEG. Twisting tests were conducted using a pair of rotatable clamps. All resistances were measured with a VICTOR LCR 4091A digital bridge. The sheet resistance of the electrode was tested from the four dimensions 280C Four‐Point‐probe mapping system. To test the output performance of uf‐TEGs, a hot plate from Thermo SCIENTIFIC was used to increase the temperature, and the voltage output and the temperature variation were both collected simultaneously from a data acquisition card of NI. The resistivity and Seeback coefficient of Bi_0.5_Sb_1.5_Te_3_ and Bi_2_Te_2.7_Se_0.3_ were measured from the commercial instrument (CTA‐3) from Beijing Cryoall Science and Technology Co., Ltd. XRD patterns were collected by an X‐ray diffractometer (Rigaku SmartLab) and the infrared image was captured by an infrared camera (Ti489 PRO FLUKE).

## Conflict of Interest

The authors declare no conflict of interest.

## Supporting information

Supporting Information

## Data Availability

The data that support the findings of this study are available from the corresponding author upon reasonable request.
