# Supplementary material for: Whole Fabric‐Assisted Thermoelectric Devices for Wearable Electronics
Source: Adv Sci (Weinh). 2021 Nov 5;9(1):2103574. doi: 10.1002/advs.202103574 (PMC8728843; doi:10.1002/advs.202103574)
Supplement: Supplementary file 1 — Supporting Information [file ADVS-9-2103574-s001.pdf]

© 2021 Wiley-VCH GmbH

ADVANCED SCIENCE
Open Access

Supporting Information

for *Adv. Sci.*, DOI: 10.1002/advs.202103574

Whole Fabric-assisted Thermoelectric Devices for Wearable Electronics

Yue Hou, Yang Yang, Ziyu Wang, Zhaoyu Li, Xingzhong Zhang, Brandon Bethers, Rui Xiong, Haizhong Guo*, Hongyu Yu**

Supporting Information

Whole Fabric-assisted Thermoelectric Devices for Wearable Electronics

Yue Hou, Yang Yang, Ziyu Wang, Zhaoyu Li, Xingzhong Zhang, Brandon Bethers, Rui Xiong, Haizhong Guo*, Hongyu Yu**

1. Fabrication Process of Textile based uf-TEGs

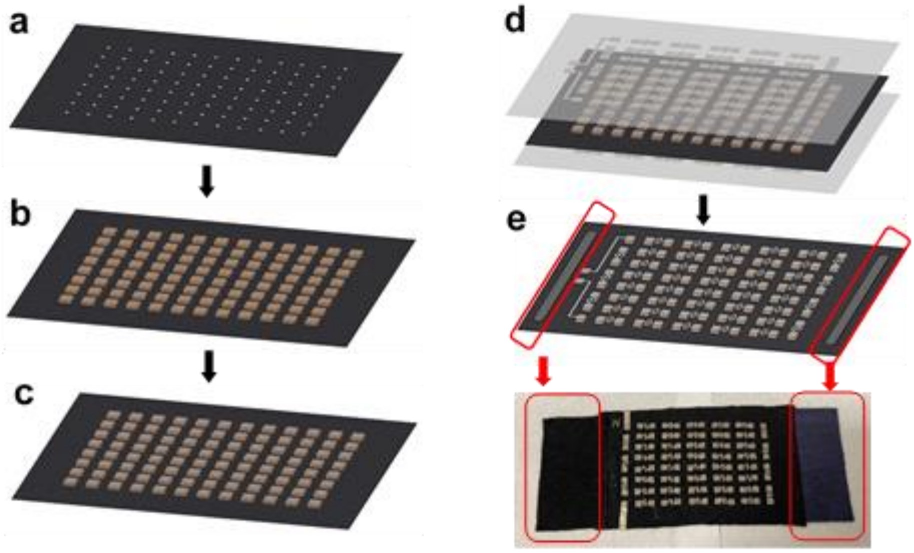

Figure S1. The fabrication process of the uf-TEGs.

Typical elastic cotton cloth was cut in a size of 150 mm × 100 mm, and the elastic cloth was laser-cut to form a circular array with a radius of 1 mm (**Figure S1**a). Then, TE cuboids were embedded in the laser-cut circles and the super glue was used to connect the TE cuboids and the cotton cloth (Figure S1b). In Figure S1c, a thin layer of solder paste was scratched on top of the TE cuboids with laser patterned acrylic molds and the PET Thermal-releasing film with the conductive electrodes array stuck on it was applied on the TE cuboids array. From Figure S1d to Figure S1e, the soldering on each side of the device was done successively on the hot plate with a temperature of 180 °C for 5 minutes. During this soldering process, a metal block was put on top of the thermal-releasing film to ensure a firm connection between the cloth electrodes and the TE cuboids. After that, the PET thermal-releasing film was connected

loosely to the cloth electrode surface, with some regions still connected due to the sticky gel of the cloth tape material after heating. Then, it could be peeled off using a tweezer under the hot gun of setting a temperature of 120 °C. As demonstrated in the red circle of Figure S1e, two mutual pastes were connected to two sides of uf-TEG. In this way, the uf-TEG can be tightly worn on arms or legs and put on cylinder-shaped heat sources like cups with good contact.

2. Testing set-up for measuring the output performance of uf-TEGs

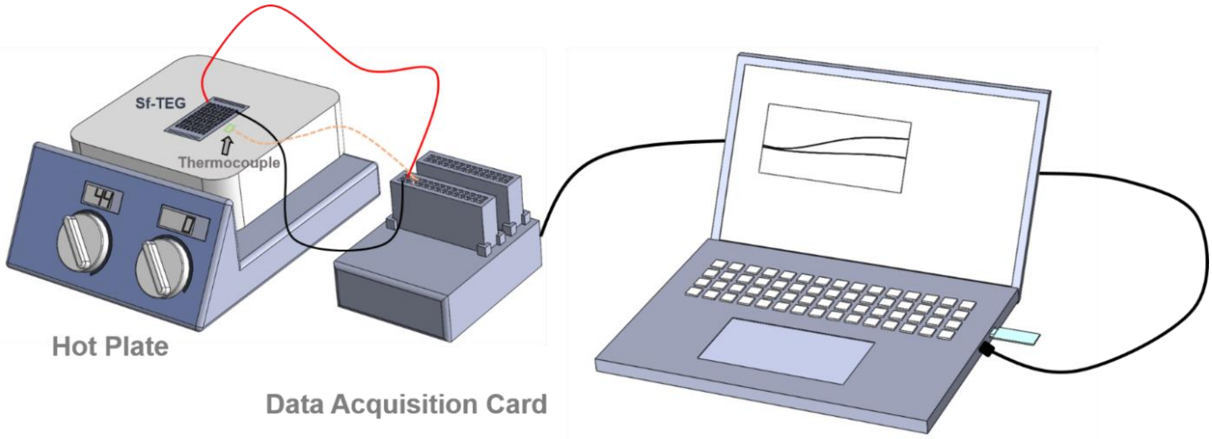

Figure S2. Testing set-up for measuring the output performance of uf-TEGs.

To test the output performance of the uf-TEG, the uf-TEG was put on top of the hot plate, the surface temperature of the hot plate was measured by a thermocouple (k type) that connected to the data acquisition card (NI). The positive and negative anodes were also connected to the data acquisition card to record the voltage output data logs. ΔT was the temperature difference of the top and bottom cuboid temperature.The temperatures on the top and the bottom of the cuboid were measured from the infrared camera.

3. Temperature distribution on different test regions

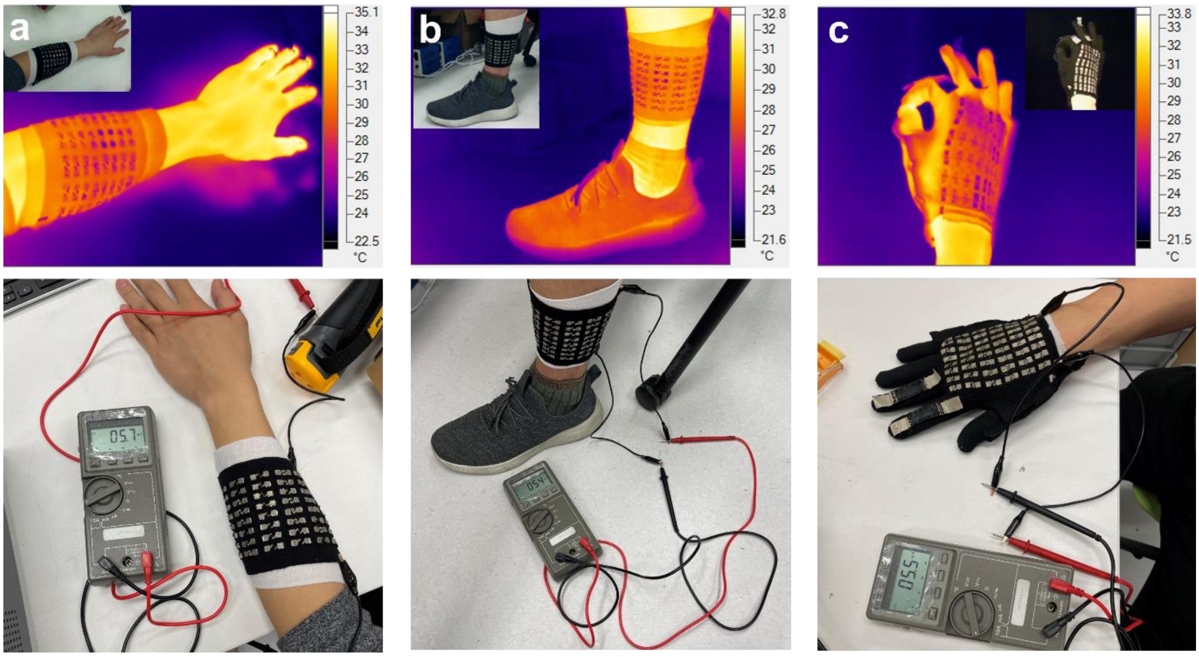

Figure S3. Temperature distribution on different regions of the human body. The infrared images and the voltage output pictures captured when the uf-TEG was worn on a) arm, b) leg, and c) hand.

Table S1. Data collection from XRD images.

	**Temperature (K)**	**Temperature after wearing a gauze (K)**	**Voltage output (mV)**
**Hand**	305.75-307.55	303.15-305.25	5.5 mV
**Leg**	304.05-305.25	302.55-304.55	5.4 mV
**Arm**	305.25-307.65	304.35-306.75	5.7 mV
**Ambient**	297.85-298.15	/	/

4. Wearable applications (wind speed affection/temperature sensing glove/human motion detection)

4.1 Wind speed affection

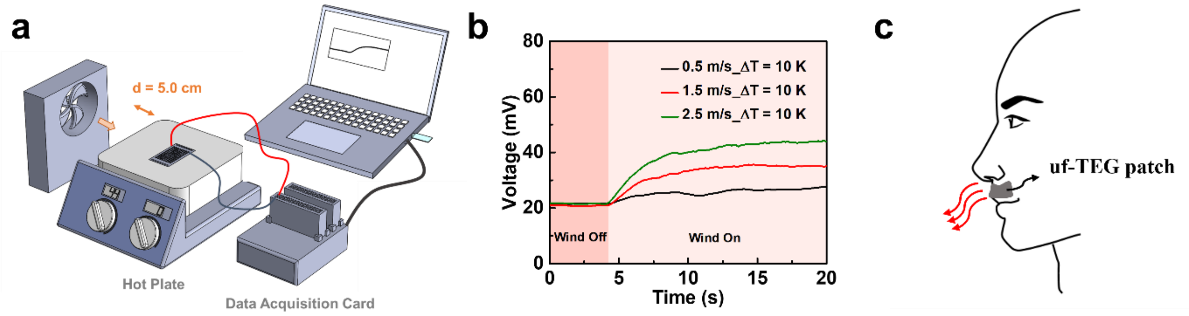

Figure S4. a) Test set-up for the wind speed affection. b) The voltage output of the uf-TEG under a wind speed of 0.5, 1.5, and 2.5 m/s. c) Nose respiration rate testing.

The effect of the wind speed on the output performance of uf-TEGs was also conducted through the experiment set-up in **Figure S4**a. The electric fan was put next to the hot plate at a distance of 5 cm and the voltage source controlled wind speed. The bottom side of the uf-TEG was set at the 35 °C (±0.5°C) with ΔT of 10 °C. The output voltage of the uf-TEG under a wind speed of 0.5, 1.5, and 2.5 m/s was recorded in Figure S4b, and the output voltage increased suddenly after opening the fan. The wind took away the heat from the upper side and increased the temperature gradient between the upper and inner sides for all TE cuboids, thereby increasing the output voltage. The nose respiration rate was measured in Figure S3c through uf-TEGs with 8 TE pairs based on the same working principle.

4.2 Temperature sensing glove

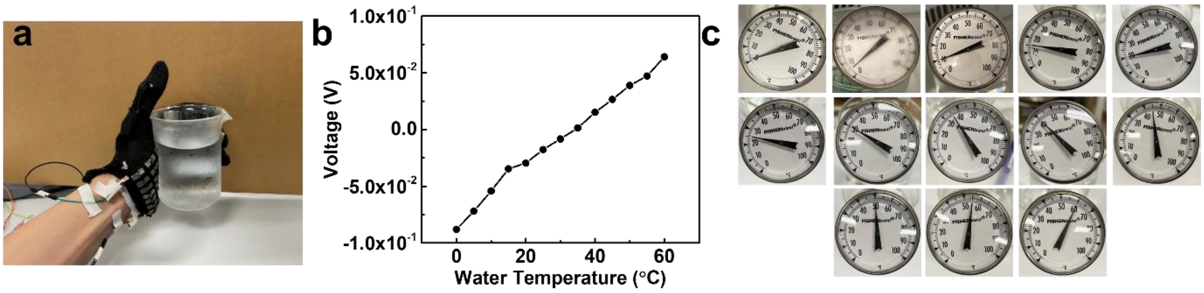

Figure S5. Temperature sensing glove testing. a) Temperature sensing glove with a beaker in hand (200 ml water). b) Temperature sensing with uf-TEG based glove (48 pairs) on hand with water temperature variated from 0°to 60°. c) water temperature in the beaker increased from 0 to 60 °C.

The temperature sensing glove was shown in **Figure S5**a. The water temperature was set from 0 to 60 °C with an interval of 5 °C and the temperature was cooled or heated by the refrigerator or hot plate. The voltage output under different water temperatures is shown in Figure S5b. The temperature was measured from the thermometer (Fisher brand) (Figure S5c).

4.3 Human motion detection

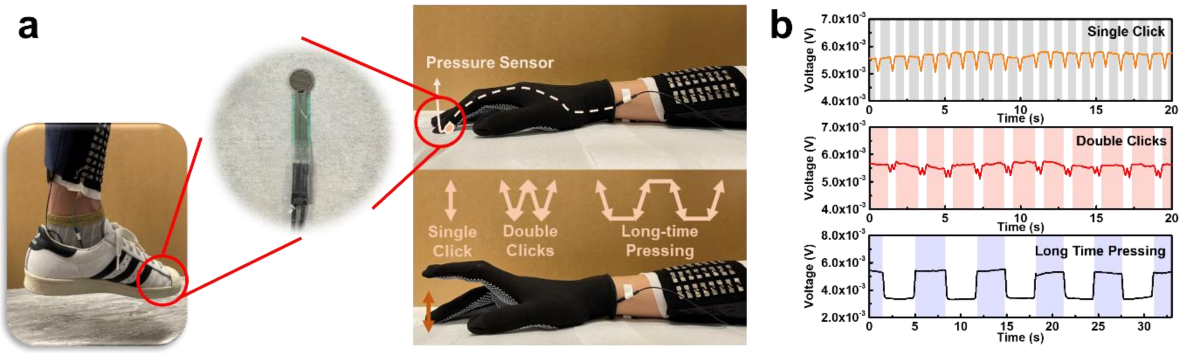

Figure S6. Human motion detection based on the uf-TEGs. a) Human motion detection based on uf-TEGs and a piezoresistive sensor. b) uf-TEG wristband (48 pairs), together with a magic glove (with a piezoresistive pressure sensor), detected the finger tapping process, including single click, double clicks, and longtime pressing movement.

A piezoresistive sensor was sewed with a sock and glove to test human motions like stepping and finger tapping (**Figure S6**a). And the piezoresistive sensor (RP-C10-LT) was purchased from LEGACT. And the finger-clicking movements (single click, double clicks and longtime pressing) are detected in Figure S6b.

4.4 Finger bending detection

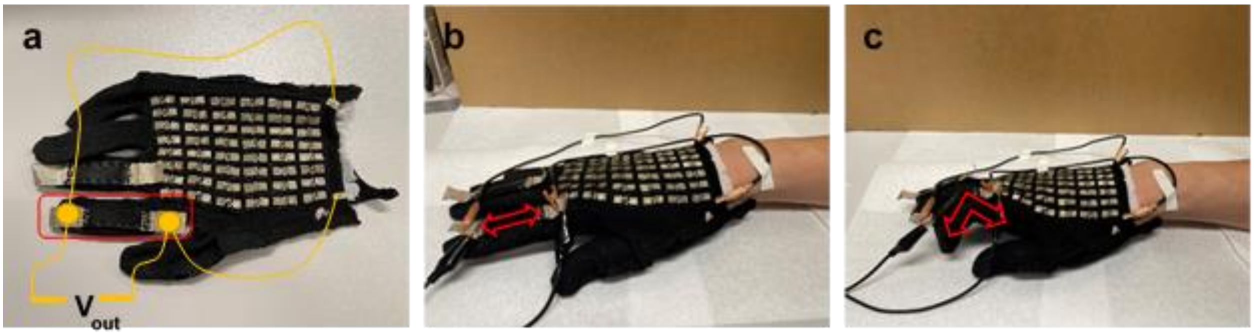

Figure S7 Finger bending detection based on the uf-TEGs. a) Testing circuit of the uf-TEG based bending sensing glove with the conductive elastomer assembled on it. Finger bending at bending angle of b) 0° and c) 90°.

The conductive elastomer with a size of 3.95 cm (± 0.05 cm) × 1.40 cm (± 0.05 cm) × 0.25 cm (± 0.05 cm) (length × width × thickness) was fabricated by mixing the multi-wall carbon nanotube (Tanfeng Tech., Inc) with Ecoflex (Smooth-on 0030) at a mix ratio of 7.0 wt% (**Figure S7**a). The conductive cloth tape was sewed on both sides of the conductive elastomer for a convenient signal readout. During the bending process, the elastomer's resistance increased, resulting in an increased load voltage readout.

5. Flexibility testing for a single electrode

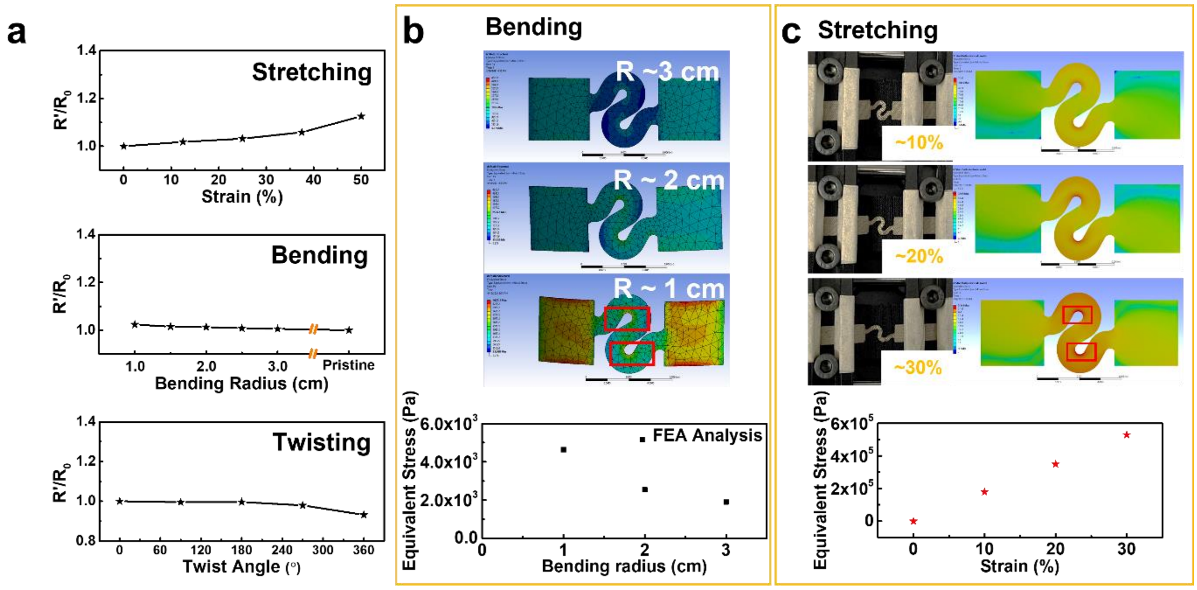

Figure S8. Flexibility testing for the polyester fiber-based electrode. a) The resistance variation (R'/R_0) during the stretching tests (under the tensile strain from 0% to 50%), bending tests (with bending radius variated from 1.0 cm to 3.0 cm, 0.5 cm interval in between) and twisting tests (with the twisting angle changed from 0° to 360°, 90° interval in between) for a single serpentine structured electrode. b) FEA simulation result of the bending test for the polyester fiber-based electrode with bending radius variated from 1.0 cm to 3.0 cm, 1.0 cm interval in between. c) FEA simulation result of the stretching test for the polyester fiber-based electrode with the strain of 10%, 20% and 30%.

To experimentally verify the electrode's flexibility and stretchability, a single electrode has been transferred on a cloth substrate to test its resistance variation under stretching, bending and twisting situations (**Figure. S8**a). As we stretched the electrode to 37.5% and 50.0 %, the corresponding resistance variations were 5.83% and 12.56%. When the electrode was bent on acrylic molds with different bending radii from 1 cm to 3 cm, with 0.5 cm intervals in between, the resistance variation was relatively small as it increased by 2.34% at the largest

bending degree (bending radius of 1.0 cm). In addition, the electrode was clamped on both side and twisted from 0° to 360°, the resistance dropped about 2.05% and 6.81% for 270° and 360°, respectively. The small resistance change during the stretching, bending, and twisting for every single electrode pave the way for the stable TE performance after assembling with TE elements on cloth substrate. FEA simulation was used to analyze the stress distribution for a single electrode with a bending radius of 1 cm, 2 cm, and 3 cm, respectively. As shown in Figure S8b, the largest stress within the connected region is located on the electrode's inner parts (pointed out in red box) with relatively small stress with three orders of magnitudes of Pascal (Pa). FEA simulation was used to analyze the stress distribution for a single electrode under the strain of 10%, 20%, and 30%, respectively. As shown in Figure S8c, the largest stress is located on the electrode's inner parts (pointed out in red rectangles), with a value of 0.53 MPa under the strain of 30%. And the stress distribution also accounted for the small folds on the top and bottom sides of the electrodes during a stretching process (Figure 4b).

6. Temperature sensing for electrical-skin application

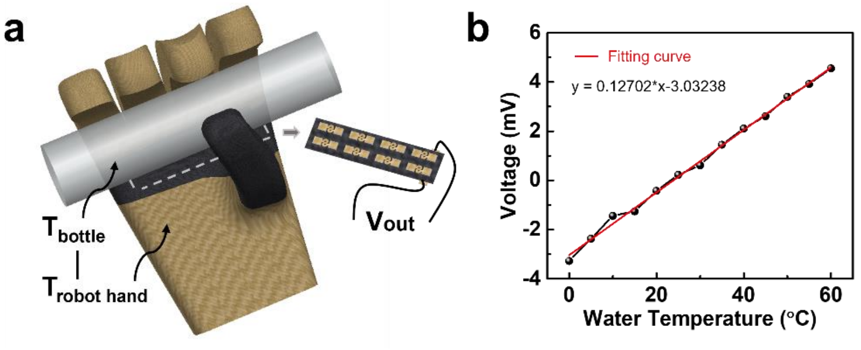

Figure S9. a) Schematic diagram of the working principle for this electrical skin of robot hand. b) Fitting result for the voltage output with the increasing water temperature.

When earing the uf-TEG as the e-skin on the robot hand, the inner temperature $T_{robot\ hand}$ was close to the ambient temperature. And the electrical skin was functional as the Temperature difference T_{bottle}-$T_{robot\ hand}$ existed when the robot hand grabbed a hot or cool

bottle (**Figure S9a**). As shown in Figure S9b, the fitting curve (red line) demonstrates good linearity for the relation between the voltage output and the input temperature difference.

7. The impact of fill factor on uf-TEGs

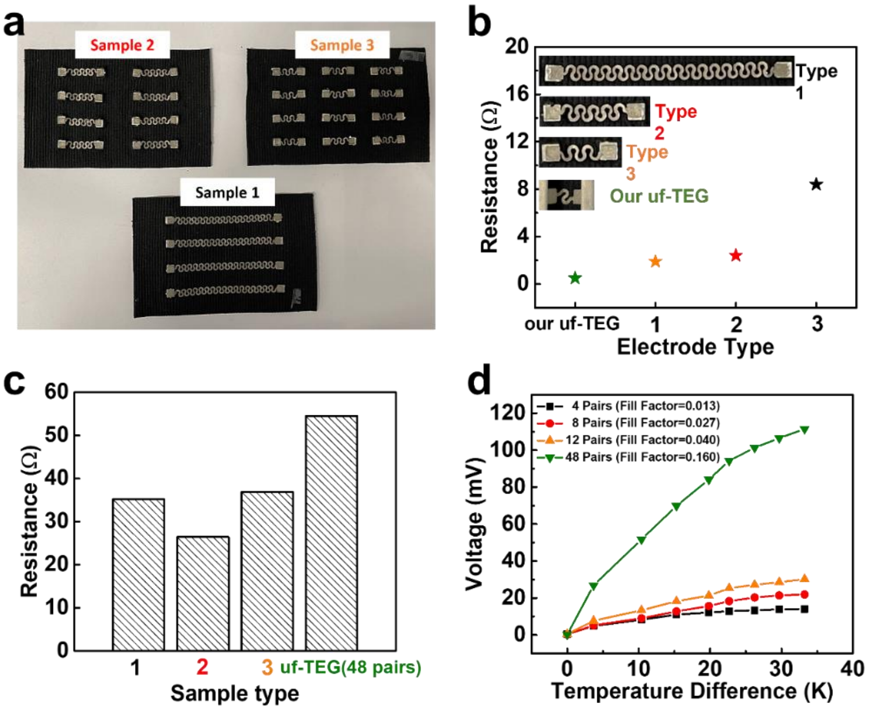

Figure S10. Uf-TEGs with different fill factors. a) Photo images of the fabricated uf-TEGs with fill factors of 0.013, 0.027, and 0.04, respectively. b) Resistance of electrodes designed for different fill factors. c) Device resistance with different fill factors. d) The output voltage of uf-TEGs with different fill factor (0.013, 0.027, 0.04, 0.16) at different ΔT.

We have conducted experiments to study the impact of the fill factor on the uf-TEG. As shown in Figure R10a, three uf-TEGs were fabricated with different fill factors. Together with the one with 48 pairs device in the manuscript, these four uf-TEGs occupied the same area but with different TE pairs. To connect them, three types of electrodes with different lengths were designed and the resistance for different types of electrodes and the whole devices were summarized in Figure S10b and S10c. These two figures illustrate that the longer the serpentine electrode, the larger the electrode resistance. In the manuscript, the resistance of a

single electrode designed for the uf-TEGs of 48 pairs is ~0.5 Ω, and the whole device resistance is ~54.5 Ω. As shown in Figure S10d, the output voltage of the 48 pairs is 3.77 times as that of the one with 12 TE pairs, but the inner resistance is only 1.48 times, according to the equation of the maximum power output ($P_{max} = \frac{V_{OC}^2}{4R_{in}}$), the P_{max} of uf-TEGs of 48 pairs is 9.60 times that of the 12 pairs, which further demonstrates that the larger the fill factor, the higher the device output power will be. Therefore, with the premise of designing a stretchable TEG, the 48 pairs device (fill factor = 0.16) with only one serpentine cycle could achieve the best device performance.

8. Stability Testing of uf-TEG

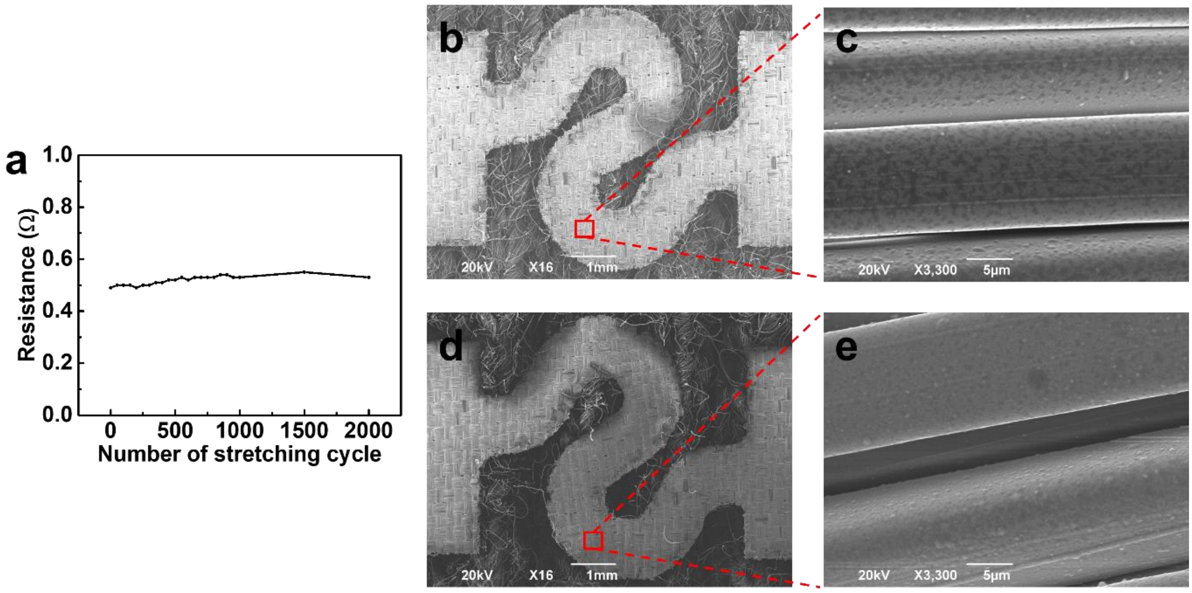

Figure S11. a) The resistance of the electrode after different bending cycles. The SEM images of the two conductive cloth electrodes: b), c) without 2000 stretching tests and d), e) with 2000 stretching tests.

The electrode resistance after different stretching cycles (from 50 to 2000 cycles) is shown in Figure S11a. After 2000 stretching cycles, the electrode resistance shows a slightly upward trend but with only 0.04Ω increase (increased by 7.1%). The SEM images were captured to show the surface morphology of the conductive fiber surface (Figure S11b and S11d).

Comparing two electrodes with and without 2000 stretching cycles (Figure S11c and S11e), especially when we focus on the largest deformation regions, we did not find any fracture on the electroplated fibers. Therefore, we speculate the woven electroplated fibers could share the localized stress, which had kept the surface metal layer from broken.

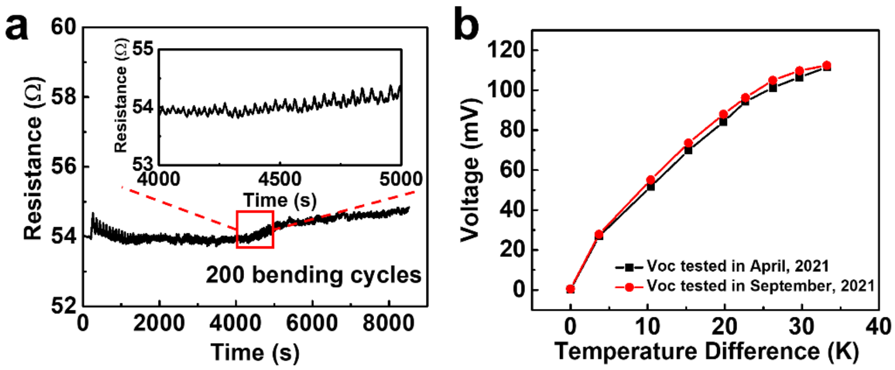

Figure S12. a) Repeated bending tests for the uf-TEGs with 200 bending cycles. b) The comparison tests for the output performance of the uf-TEG conducted in April and in September 2021.

The repeated bending test was also conducted by testing the device resistance under the variate bending radius from ∞ to 2.5 cm for 200 cycles. The device with soft cloth electrode and fabric connection was flexible enough for this repeated bending test. The results in Fig. S12a also show a good long-term bending stability with the resistance variation only increase for 1.52% after the 200 cycles. We have further tested the open-circuit voltage at different ΔT for the same uf-TEGs in Sep 2021, and the testing result is compared with the one in Apr 2021 (Figure. S12b). The device was not stored in the vacuum chamber in the past five months, and from the test result, we did not find any performance degradation.
